# A Methodological Framework to Study Change in Team Cognition Under the Dynamical Hypothesis

**DOI:** 10.1111/tops.12685

**Published:** 2023-08-29

**Authors:** Kyana van Eijndhoven, Travis J. Wiltshire, Elwira A. Hałgas, Josette M. P. Gevers

**Affiliations:** ^1^ Department of Cognitive Science and Artificial Intelligence Tilburg University; ^2^ Department of Industrial Engineering and Innovation Sciences Eindhoven University of Technology

**Keywords:** Dynamical systems, Team coordination, Team cognition, Team adaptation, Team psychophysiology, Recurrence quantification analysis

## Abstract

The dynamical hypothesis claims that cognitive systems, such as teams, are dynamical systems (i.e., an interdependent collection of individuals and their technology that change together over time). Following this hypothesis, team researchers have adopted dynamical approaches to better understand the team cognitive processes and states that form *team cognition*, as well as how they emerge over time. One approach focuses on *team coordination dynamics*, which examines the coupling of signals between interacting individuals in various modalities, and has been shown to reflect aspects of team functioning including team cognition. However, how changes in team coordination relate to high‐level team cognitive processes and states, as well as important events, are not yet fully understood. To this end, we advance a methodological framework for researching team cognition under the dynamical hypothesis. Subsequently, we provided an empirical case‐study application of this framework. Thereby, this work contributes methodologically and empirically to a deeper understanding of team cognition, the dynamical hypothesis, and the synergy between them.

## Abstract 2

We advance a methodological framework for investigating how changes in team coordination relate to high‐level team cognitive processes and states, as well as important events. We demonstrate the framework with an empirical case‐study. This work contributes to a deeper understanding of team cognitive science, the dynamical hypothesis, and the synergy between them.

## 1 Introduction

Team cognition is vital to the performance of teams operating under highly complex and stressful circumstances, such as military or healthcare teams (DeChurch & Mesmer‐Magnus, 2010b). Consequently, many efforts have been made to gain a deeper understanding of this multilevel construct. Findings from previous research indicate that processes and states comprising high‐level team cognition are fundamentally dependent on their observable, low‐level coordination dynamics (Cooke, Gorman, Myers, & Duran, 2013; Gorman et al., 2010; Gorman & Amazeen, 2010). *High‐level cognition* consists of goal‐directed processes and states involving multiple cognitive subsystems to process, interpret, exchange, and reframe information (Ragni & Stolzenburg, 2015). Multiple high‐level team cognitive processes such as team monitoring (Haataja et al., 2018) and team adaptation (Gorman et al., 2020), and states such as those related to cognitive workload (Ahonen et al., 2016) and mental effort (Dindar, Järvelä, & Haataja, 2020), have been linked to specific patterns in team coordination dynamics. Such dynamics can be considered “low‐level” because they often occur unintentionally between team members (Knoblich, Butterfill, & Sebanz, 2011) and they tend to occur at smaller scales of analysis when compared to an entire team (e.g., physiology, motor control, etc.; Fusaroli, Rączaszek‐Leonardi, & Tylén, 2014).

*Team coordination dynamics* represent the coupling of signals between interacting team members that occur across multiple modalities (e.g., movements, physiology, and speech), to address changes in the team's working environment. Specific patterns of signal couplings can be, for example, synchrony (a systems’ components change simultaneously and consistently) or recurrence (a systems’ components revisit pattern sequences). Such patterns, also referred to as states within coordination dynamics, can be observed across a variety of signals (for an overview, see Hałgas et al., 2022; Kazi et al., 2021; Palumbo et al., 2017). For example, synchrony in heart rate variability has been linked to performance speed of surgical teams (Powezka et al., 2022) and recurrence in electrodermal activity (EDA) has been related to collective mental effort (Dindar et al., 2020).

Moreover, transitions between team coordination states are found to be indicative of changes in the high‐level cognitive processes and states occurring in teams (Amazeen, 2018; Likens, Amazeen, Stevens, Galloway, & Gorman, 2014). However, the ways in which changes in high‐level team cognition relate to changes in the low‐level coordination are not yet fully understood. In addition, there is a lack of studies examining how those changes at different levels of team cognition relate to events within teamwork (e.g., episode of ineffective team functioning; cf., Gorman et al., 2020, 2016). Such knowledge is crucial in gaining a deeper understanding of team cognition, and can subsequently enable the support of high‐level cognitive team processes and states based on team coordination dynamics.

### 1.1 Team cognition theory and the dynamical hypothesis

Research regarding team cognitive processes is often broadly motivated by two distinct theoretical frameworks (Wildman, Salas, & Scott, 2014). On the one hand, information processing‐based theory of team cognitive processes have focused on static and linear interpretations, such as knowledge structures (e.g., Gualtieri, Burns, Phipps, Reeves, & Pierce, 1998), input‐process‐output models (e.g., McGrath, 1964), or computational‐representational brain‐based processes (e.g., Pylyshyn, 1989). On the other hand, researchers have advanced team cognitive theory from a more dynamical perspective, interpreting team cognition as continuously changing over time in response to their environment. Unlike the more traditional theories, this perspective shifts the focus from the individual cognitive structure to the systemic team‐level, and does not consider teams simply as the sum of their parts (Cooke et al., 2013), but also emphasizes key dynamical systems properties.

Team cognition research, from this dynamical perspective, has developed consistent with *the dynamical hypothesis*. This hypothesis claims that cognitive systems, such as teams and their individual and collective cognitive processes and states, are dynamical systems (van Gelder, 1995, 1998; van Gelder & Port, 1995). Put differently, cognitive systems, consisting of multiple interacting components that change over time and across multiple levels to adapt to the system's environment, are *dynamical systems* (Kelso, 1994). Components within dynamical systems (e.g., team members within a team) interact with each other, forming networks of interaction that contribute to the patterns characterizing the systems’ behavior. As this process occurs spontaneously, and without a central and external controller, dynamical systems are said to self‐organize into system‐level behaviors due to, and constraining, component‐level interactions (Kelso, 1994). According to the dynamical hypothesis, the presence of self‐organization is one of the critical aspects as to why cognition should be addressed from a dynamical perspective (van Gelder, 1998).

In addition to change over time across multiple levels and self‐organization, cognitive systems have also been found to exhibit other key properties of dynamical systems (e.g., see Gorman, Dunbar, Grimm, & Gipson, 2017; van Gelder & Port, 1995), such as multistability. Ranges of stable values (e.g., of team coordination), which occur more regularly, represent stable states, of which multiple tend to be present for dynamical systems. When dynamical systems experience perturbations, they often return to stable states (Kelso, 2012). The identification of these states could, for example, enable the exploration of what patterns in team coordination dynamics occur while the system resides in such states, and how those patterns relate to high‐level team cognition and team events. Consequently, assessing how different key properties are exhibited in the specific context of team cognition as a dynamical system can provide researchers with deeper insights of change at multiple levels of team cognition.

### 1.2 Empirical work on team cognition

While the knowledge‐based approaches to team cognition have shown great utility in predicting task performance and other aspects of team functioning (Bui, Chau, Degl'Innocenti, Leone, & Vicentini, 2019; DeChurch & Mesmer‐Magnus, 2010a, 2010b), it is not well suited for understanding how the real‐time interactions of team members, and their coordination dynamics, contribute to such high‐level cognitive processes and team outcomes (Hałgas et al., 2022; Wildman et al., 2014). However, empirical work in this area is challenging because it requires the integration of theoretical advances like the dynamical hypothesis, with investigative frameworks that scaffold empirical methodologies. On the one hand, empirical work regarding team cognition, for example, has examined how teams exhibit a variety of characteristics commonly found in dynamical systems (Gorman et al., 2017), such as movement (Wiltshire, Steffensen, & Fiore, 2019) and neural synchronization (Stevens, Galloway, Wang, & Berka, 2012), phase transitions (Wiltshire, Butner, & Fiore, 2018), communication reorganizations (Gorman et al., 2020), and coordination breakdowns (van Eijndhoven, Wiltshire, Hałgas, & Gevers, 2023). Importantly, these dynamical properties are related to various aspects of team functioning (Hałgas et al., 2022). With regard to integrative frameworks, Favela (2020) constructed an investigative framework to address questions in cognitive science by approaching cognition through the lens of dynamical systems. In addition, Wiltshire, van Eijndhoven, Halgas, and Gevers (2022) constructed a framework for augmenting adaptive team performance by monitoring team coordination dynamics underlying team cognition and providing feedback. Thus, a variety of dynamical approaches have been developed to examine different levels of team cognition. Yet, while some frameworks exist, there have been little step‐by‐step demonstrative applications of them; And in particular, none that combine the examination of change within team cognition at different levels, and examine those changes with regard to each other as well as events in teamwork (c.f., Gorman et al., 2020).

### 1.3 Aims of the current work

We aim to provide such an investigative framework, which enables examining team cognition from a dynamical perspective, and helps further deepen its integration with team cognitive science. To this end, we adapt the framework by Wiltshire et al. (2022), expanding the focus on change in team coordination, to include the assessment of change in high‐level team cognition and events in teamwork. The framework steps include: identifying low‐ and high‐level team cognition variables of interest, examining their trajectory over time, identifying change at different levels of cognition and locating team events, and combining this information for joint examination.

Similar to previous case studies (e.g., Clark, Cott, & Drinka, 2007; Furniss, Back, Blandford, Hildebrandt, & Broberg, 2011; Ismail, Khelifi, & Harous, 2022), to illustrate each step within the framework, we utilize a small data sample. Accordingly, the findings we describe in the current paper are not intended as broader conclusions. We do, however, contend that such conclusions could be generated with our framework using larger data samples. Nonetheless, the examples here demonstrate how this framework could be used to generate insights regarding team cognition, and the functionality of the dynamical hypothesis for assessing team cognition. As with most case studies, this work provides in‐depth examples to understand the process, which can be useful for the development of new research questions and is complementary to statistical methods that could be applied on a larger sample (Flyvbjerg, 2011).

## 2 Data sample description

The data sample utilized in the current paper was part of a larger project (van Eijndhoven et al., 2023). This project involved data collection from 47 four‐person teams playing Lovers in a Dangerous Space Time (Hammill, Tucker, & Winkels, 2015), which is a collaborative and challenging game that can be stress‐inducing for teams. Team members were placed on a spaceship, and assigned specific roles (e.g., steering the ship) creating interdependencies. Their goal was to save at least five abducted nonplayable characters from enemies, and to finish the level before their ship health ran out. Teams played a tutorial and three levels on computers organized in a 2 (side‐by‐side) × 2 (face‐to‐face) setup, ensuring members could see each other and communicate. During this task, EDA and photoplethysmogram (PPG) data were captured with a Shimmer GSR+ wearable sensor (Burns et al., 2010). In addition, the faces of participants were recorded with a webcam, audio with headset microphones, and the gameplay was recorded with OBS Studio software. A detailed description of this larger dataset can be found on the Open Science Framework (https://osf.io/t9eym), as well as the current materials (https://osf.io/tsxcq) and code (https://osf.io/x2f56).

To provide a practical example of each step of our framework, we utilize data of two teams (each consisting of two women and two men; M_age_ = 21; age range = 19–24). More specifically, we estimate team coordination based on PPG data from each team member as low‐level team cognition, and team adaptation based on behavioral annotations as high‐level team cognition (see Step 1). We focus on data collected from level three of the game. This level was selected, because an experimental manipulation was introduced where the experimenter disabled one player's controls, and told the team that there were technical issues. These teams were required to play the level with only three, instead of four players (perturbation condition), though communication between all four members was still possible. That level also had a different layout than previous levels, requiring adaptation. The teams from the perturbation condition were chosen to illustrate those that are adaptive to this scenario and those that were not (i.e., reflecting the high‐level team cognition variable of interest) based on a set of criteria. Further details regarding these criteria are discussed throughout the framework (or here: https://osf.io/pmbc7).

## 3 A framework to study team cognition under the dynamical hypothesis

### 3.1 Step 1: Identify the team cognition variables of interest

In this step, we discuss low‐ and high‐level team cognition. In particular, we consider several relationships that have been examined between low‐level team coordination and high‐level processes and states, and how different measures capture coordination. Building on this literature, we suggest considerations for identifying team cognition variables. Finally, we provide a description of two team cognition variables, which will be referred to throughout the steps. These variables are utilized to illustrate an implementation of the framework. Subsequently, again, the findings we describe are not intended as broader conclusions, but are meant to show readers how to interpret the information generated through the steps. With Step 1, we aim to provide considerations for identifying team cognition variables of interest, and show how this identification can be carried out.

In previous literature, team cognition has been established as a multilevel phenomenon (DeChurch & Mesmer‐Magnus, 2010b). While there are numerous examples of low‐level team cognition processes and states (e.g., joint attention, team working memory; Cuevas, Fiore, Caldwell, & Strater, 2007), when it comes to change in team cognition, team coordination has frequently been assessed. Interactive Team Cognition theory (Cooke et al., 2013) proposes that team cognition resides in team coordination, through interactions of information exchange, and emphasizes the role of coordination in adapting to changes in the environment. This theory connects closely to the dynamical hypothesis (van Gelder & Port, 1995), which states that cognition takes place over multiple simultaneous interactions occurring at different timescales (e.g., heartbeat relating to smaller timescale, problem‐solving relating to larger timescale). Moreover, a multitude of studies found team coordination to reflect high‐level team cognition changes (e.g., Gorman & Amazeen, 2010; Gorman et al., 2020; van Eijndhoven et al., 2023). Given these theoretical and practical findings, the current framework suggests an assessment of team coordination as low‐level team cognition.

However, different signals reflecting team coordination have been observed to relate to different high‐level team cognition processes and states (Hałgas et al., 2022; Kazi et al., 2021). Examples of such relationships are in Table 1.

**Table 1:** Overview of a selection of signals reflecting team coordination and how they were linked to high‐level team cognition

Signal	Signal description	Relationship to high‐level team cognition	Source
Communication	Turn‐taking time‐series of speakers	Turn‐taking in team speech predicted team reorganizations to adapt to perturbations	Gorman et al. (2020)
Movement	Level of overall bodily movement	Movement coordination reflected performance of collaborative tasks	Wiltshire et al. (2019)
Physiology	Heart rate variability (HRV)	Coordination in HRV indicated team cognitive load	Dias, Zenati, Stevens, Gabany, and Yule (2019)
Neurophysiology	Electroencephalography (EEG)	EEG coordination indicated team adaptiveness and flexibility	Dodel, Tognoli, and Kelso (2020)

Table 1 also indicates that while the choice of signal is an important factor regarding these relationships, the measures of coordination that translate team members’ signals into a team‐level reflection of coordination (i.e., team coordination dynamics) are also of significance. Examples of such measures are recurrence quantification analysis (Webber & Zbilut, 1994), cluster phase synchrony (Frank & Richardson, 2010), and pattern entropy (Stevens et al., 2012). For an overview of coordination measures, see Hałgas et al. (2022) and Wiltshire et al. (2022), as well as Hudson, Wiltshire, and Atzmueller (2022) for the relationship between them.

Previous studies suggest that different combinations of signals and measures of coordination may capture unique team cognitive processes and states (Hałgas et al., 2022; van Eijndhoven et al., 2023; Wiltshire, Steffensen, & Likens, 2020). Researchers should take this into consideration when identifying team cognition variables of interest. To make decisions regarding signals, measures, and team cognitive processes and states, previous team literature is a valuable source of information (e.g., Hałgas et al., 2022; Kazi et al., 2021; Palumbo et al., 2017). Additionally, a more exploratory approach can be taken, by including a variety of signals, measures, and team cognitive processes and states.

To illustrate our framework's steps, we provide an example based on a small sample of team coordination data derived from PPG data (heart rate) with multidimensional recurrence quantification analysis (MdRQA). The teams’ PPG time‐series were embedded into a multidimensional phase space representing the systems’ coordination dynamics. States of the embedded time‐series are identified as recurrent, if their similarity falls within a radius threshold. Multiple properties reflecting different aspects of recurrence can be quantified (e.g., see Wallot, Roepstorff, & Mønster, 2016). The current example focuses on determinism (%DET), which estimates the regularity of recurrent sequences of states. This time‐series represents the low‐level team cognition metric (see van Eijndhoven et al., 2023 for details).

**Fig. 1:**
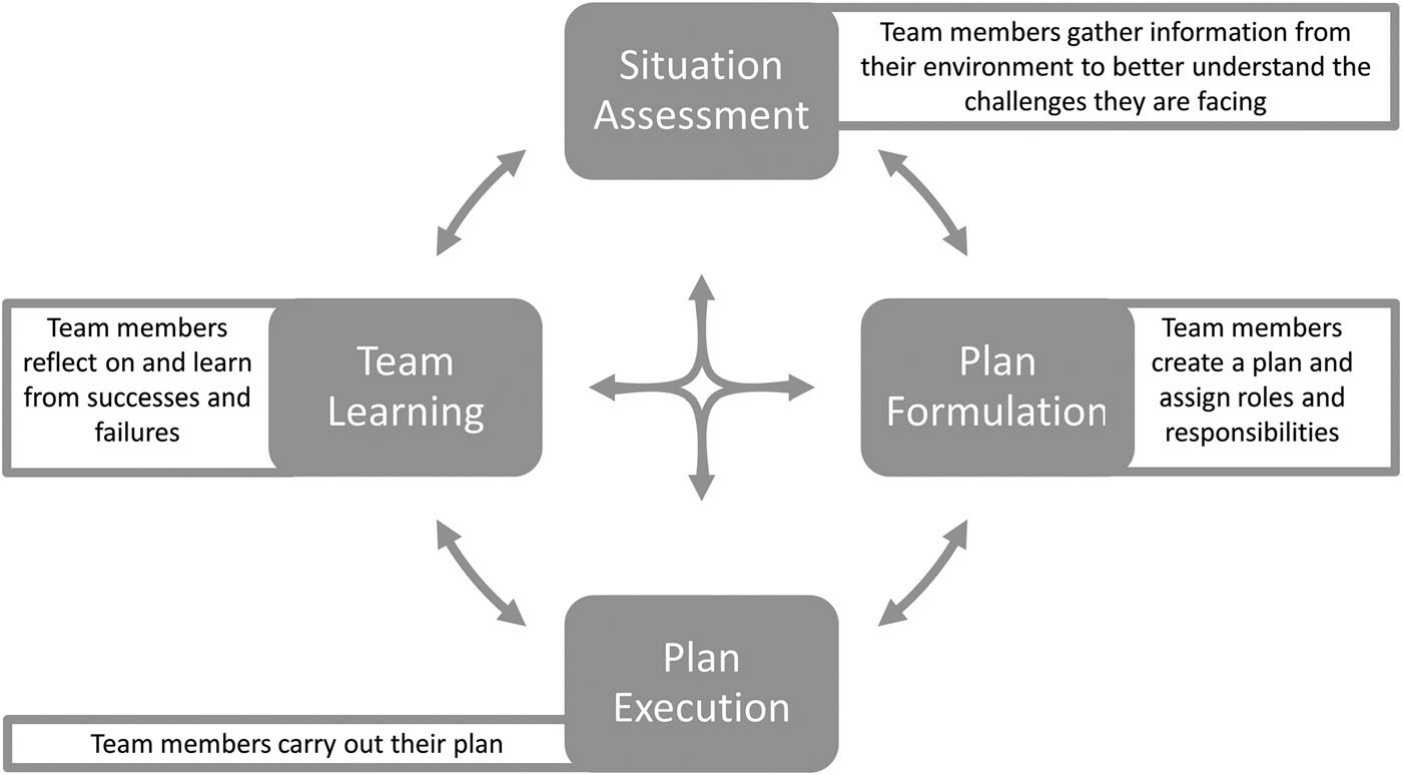
Phases of team adaptation (Burke et al., 2006) used for annotation.

The high‐level metric reflects the team adaptation process. *Team adaptation* was operationalized following Burke, Stagl, Salas, Pierce, and Kendall's (2006) definition: an emergent and dynamical phenomenon spanning multiple phases (see Fig. 1), during which team members functionally rearrange their behavior to respond to changes in task demands. Annotations for each of these team adaptation phases were made utilizing Adobe Audition software (version 23.2) using the webcam and gameplay recordings. To illustrate two different examples of the team adaptation process, we applied our framework to one maladaptive team that unsuccessfully responded to perturbation (i.e., did not complete game level), and one adaptive team that was successful (completed game level). Both teams were from the perturbation condition. The maladaptive team was chosen at random from a list of teams that did not complete the game level. While six teams in the perturbation condition completed the game level, data for only one team were suitable for further analysis (due to data quality issues regarding physiological data or incomplete audiovisual data). This team was subsequently chosen as the adaptive team.

The phases in Fig. 1 have previously also been used as the base of a team adaptation annotation scheme by Georganta and Brodbeck (2020). However, while their scheme was designed for a more slow‐paced task, our data were based on a fast‐paced collaborative game. Therefore, we interpret the adaptation phases as order independent. Moreover, only general phases of team adaptation were annotated throughout the recordings. Additionally, team activities that did not qualify as any of the team adaptation phases were annotated as “Regular gameplay”, and the phase during which the external perturbation was introduced (the team was forced to play with three instead of the expected four members) was annotated as “External perturbation.”

### 3.2 Step 2: Examine the trajectory of team cognition at different levels

**Fig. 2:**
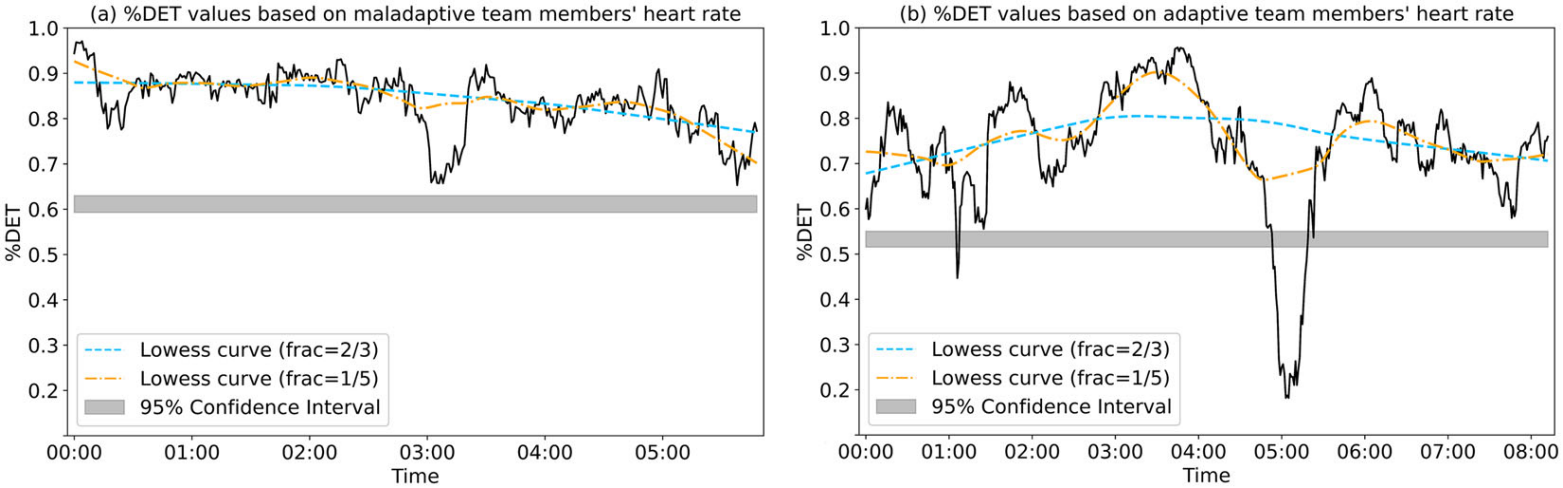
Maladaptive (Plot a) and adaptive (Plot b) teams’ time‐series plots of low‐level team cognition metric, Lowess curves reflect general trend within data at different levels of detail, 95% confidence intervals were calculated by bootstrapping heart rate values (1,000 iterations per team), calculating %DET, and deriving the averaged confidence intervals. Frac reflects the size of the sliding window (as a ratio of the whole dataset) used to estimate Lowess curves.

In Step 2, we provide suggestions on how to examine team cognition trajectories, and show an implementation of these suggestions utilizing our sample data. To better understand change in team cognition through the chosen variables, it is insightful to visualize their trajectories. With a multitude of strategies available (see, e.g., Xu, de Barbaro, Abney, & Cox, 2020), Wiltshire et al. (2022) suggest time‐series plots as a straightforward means to get insight into team coordination dynamics. Utilizing our running examples discussed in Step 1, Fig. 2 displays time‐series plots of %DET values based on maladaptive and adaptive team members’ heart rates.

The %DET trajectories displayed in Fig. 2 were calculated with MdRQA on each team member's heart rate, which is a measure that quantifies the regularity of recurrent sequences of states ranging from 0 to 1 (see Step 1). For the maladaptive team displayed in Plot a, %DET values fall between 0.65 and 1, indicating that a majority of recurrent points form longer recurrent sequences, and that the %DET variable tends to gravitate toward higher stability of recurring patterns. The %DET time‐series of the adaptive team (Plot b) shows greater variation with values between 0.18 and 0.96, suggesting both highly stable and unstable recurrence sequences. This range difference could indicate that adaptive teams have more flexibility in response to environmental changes, and thus a larger range in %DET. Maladaptive teams on the other hand could be seen as more rigid, with a smaller %DET range. In addition, the Lowess curves in Plot a reflect a general downward trend, indicating a decrease in stability, whereas those in Plot b indicate a slight upward trend (stability increase) followed by a slight downward trend (stability decrease). Finally, the majority of %DET values was outside of the 95% confidence intervals, indicating that we observed greater than chance %DET values.

**Fig. 3:**
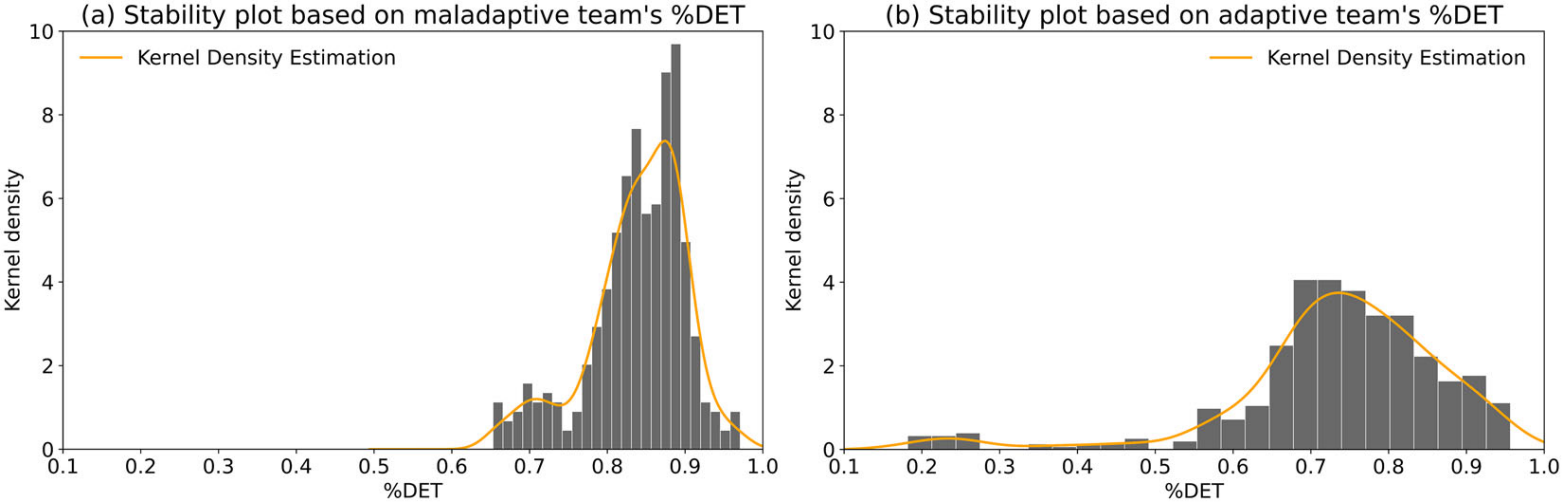
Stability plot example of low‐level team cognition variable for maladaptive (Plot a) and adaptive (Plot b) team.

Related to the trajectory of the team coordination dynamics is the stability of its values. One can visualize the stability of coordination states by examining the density of values with higher densities corresponding to more stable states (see Fig. 3).

Plot a displays a small peak in density at around .71 %DET, and a larger peak at around .87. Plot b shows a small peak at around .23 %DET, and a larger one at around .73. This could indicate multiple stable states (multistability; a key property relating to the dynamical hypothesis), with higher %DET values more resilient to perturbations (stronger stability) than lower values (weaker stability).

**Fig. 4:**
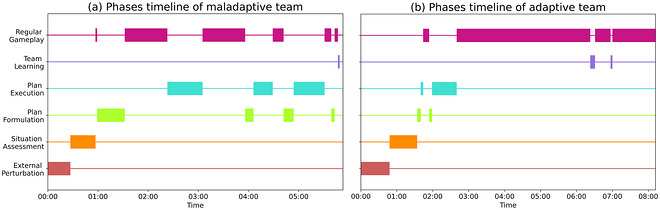
Visualization example of high‐level team cognition metric regarding unsuccessful (Plot a) and successful (Plot b) team adaptation.

Fig. 4 shows the changes between the annotated external perturbation, regular gameplay, and team adaptation phases over time. Both plots in Fig. 4 show that after the perturbation was introduced, the teams assessed the changed situation. While the maladaptive team then went through multiple sequences of plan formulation, plan execution, and regular gameplay throughout the game level, the adaptive team only did so during the first half. Finally, team learning occurred very briefly for the maladaptive team, right before they failed the level. Team learning for the adaptive team took place twice, earlier in the level. Overall, in line with the dynamical hypothesis, the visualizations in Step 1 show that the teams’ cognition changes over time at multiple levels. Low‐level team coordination was shown to follow different trajectories (Fig. 2) and exhibit different stable states (Fig. 3). High‐level team adaptation phases varied over time (Fig. 4).

### 3.3 Step 3: Identify change within low‐level team cognition and locate team events

Step 3 involves the identification of change in team coordination and location of team events. Subsequent results will be demonstrated and interpreted in combination with previously gathered information for Step 4. Various methods that are available to identify change are applicable to team coordination dynamics (e.g., see Aminikhanghahi & Cook, 2017). A brief overview of example methods is provided in Table 2.

**Table 2:** Overview of a selection of methods suitable for identifying change

Method type	Computation	Change identification	Application examples
Entropy‐based methods	Entropy values representing the amount of disorder within a system	Peaks in entropy values	Stevens & Galloway, 2019; Wiltshire et al., 2018
Dynamic complexity‐based methods	Values of fluctuation intensity and distribution randomness	Computed values exceed given threshold	Olthof et al., 2020; Schiepek & Strunk, 2010
Change point algorithms	Comparison of probability distributions of data	Significant difference between two distributions	Killick, Fearnhead, & Eckley, 2012; Taylor, 2000; Wang & Fan, 2021
Nonlinear prediction algorithms	Predicted trajectory values obtained through a nonlinear model trained on subset of data	Deviations between predicted and actual trajectories exceeding threshold	Gorman et al., 2020

Each method described in Table 2 provides a different approach to identifying change. Similar to Step 1, researchers should refer to previous literature on how change in the chosen team coordination variable was identified, to find a suitable method. Alternatively, a variety of methods can be included to further investigate which are appropriate.

A nonlinear prediction algorithm (NLPA), mentioned in Table 2, was applied to the example team data. More specifically, we applied a sliding window version of the NLPA (window size = 42 s, window step = 1 s). At each window step, the team coordination data within this window were split into two parts. A nonlinear model with S‐Map was generated based on the first half of each window of data (Sugihara & May, 1990). With S‐map, data are mapped into a phase space and a predictive model is built using nearest neighbors. The model attempts to predict the trajectory of the second half of each window of data. Root mean square error (RMSE) was computed to evaluate the differences between the predicted and actual trajectories, with a threshold for identifying change set at two standard deviations away from the mean RMSE (Hawkes & Webb, 1963). The identification of such changes is important, as previous studies found they can relate to changes in high‐level team cognition. An example of how to interpret these changes at different levels is provided in Step 4.

For Step 3, we also located team events. Team cognition is driven by changes in the team's environment (Interactive Team Cognition theory; Cooke et al., 2013, dynamical hypothesis; van Gelder & Port, 1995), and subsequently, it is important to take team events into considerations when assessing change in team cognition. In general, at least two types of team events should be annotated: (1) events that induce change in the previously chosen high‐level team cognition metric, and (2) events of achievement or failure that relate to the previously chosen high‐level team cognition. In our case, we were interested in team adaptation. Therefore, we annotated (1) the moment teams first encountered novel situations that required a response involving team adaptation, and (2) the achievement or failure of a joint goal requiring team adaptation. Team events annotated for the former involve the team encountering a wave of enemies that should be eliminated in a novel environment. An example of the latter involves the coordination between team members to simultaneously hit a target (button), which is required for the team to advance further in the game. In Step 4, we provide an assessment of how changes at different levels of team cognition relate to these identified events.

### 3.4 Step 4: Examine change at different levels of team cognition and team events

**Fig. 5:**
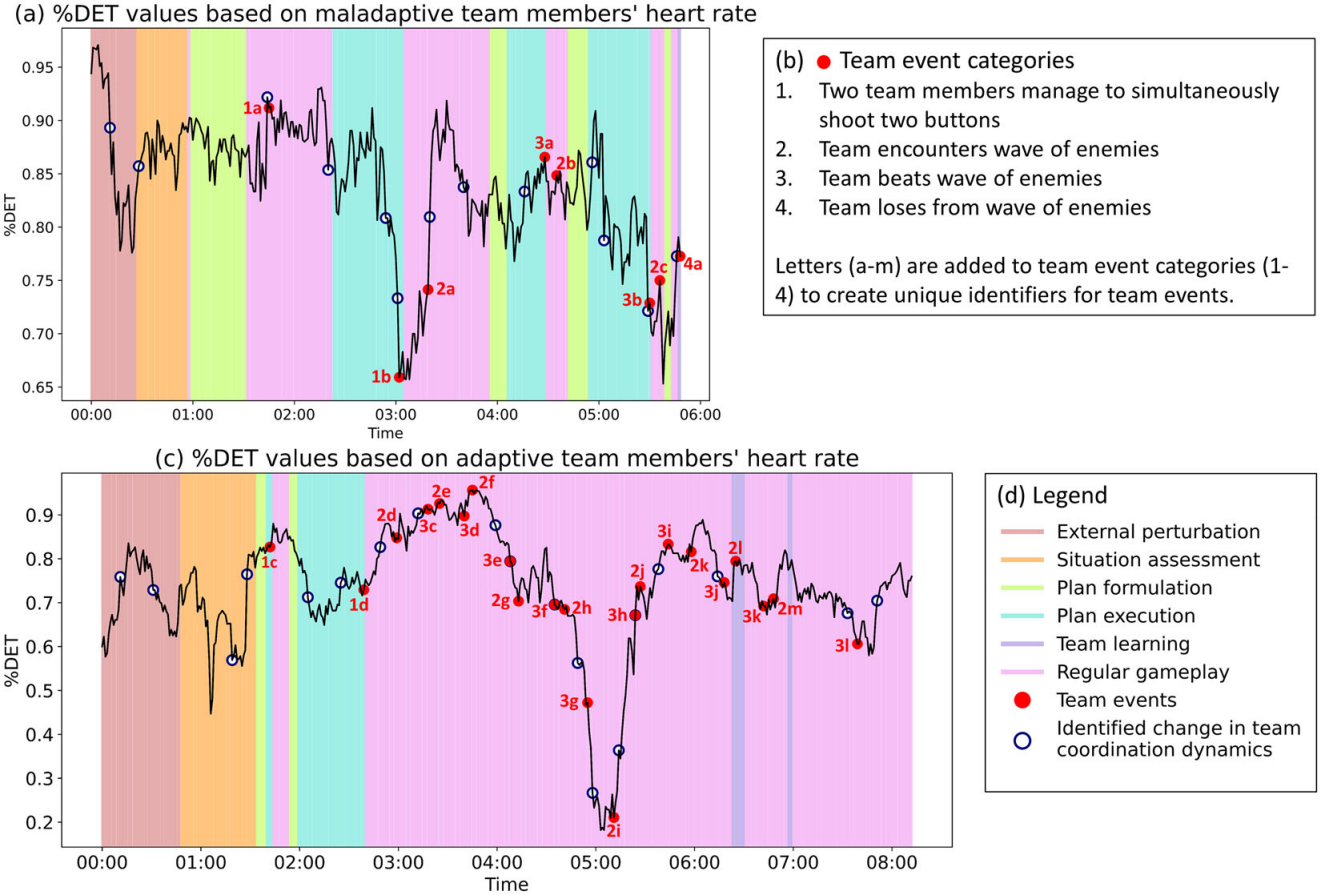
Visualization of different levels of team cognition and team events for maladaptive (Plot a) and adaptive (Plot c) teams, an overview of team events to which letters are added to create unique team event identifiers (Plot b), and a legend (Plot d). Note that axes are different so interpretation of the relative size of changes between the two teams should be done with caution.

With this framework, we aimed to (1) examine team cognition at different levels under the dynamical hypothesis, (2) examine changes in team cognition at different levels with regard to each other, and (3) examine those changes with regard to events in teamwork. Team cognition at different levels was previously assessed through Steps 1–3 of our framework. To facilitate our examination regarding aims 2 and 3, in Step 4 we generate a visualization combining the information that was gathered in previous steps, to display the team cognition metrics as well as team events, and changes therein (see Fig. 5). Visualizing all components together, can help researchers to examine (1) how changes in team cognition at different levels relate, (2) how team events relate to low‐level team cognition changes, (3) and high‐level team cognition changes, and finally, (4) we consider the combination of change within team cognition at different levels and team events. To demonstrate this process, we show such a joint visualization, and discuss our qualitative observations.

To study how changes in team cognition at different levels relate, we examine both the blue circles on the %DET line, that were identified as points of change in team coordination using the NLPE algorithm, as well as the change of team adaptation phases, with each color representing a different adaptation phase. An interesting observation is that for the maladaptive team (Plot a), change from the external perturbation phase to the situation assessment phase, and from regular game play to the team learning phase coincided with an identified change in team coordination. For the adaptive team, however, this was only the case for change from situation assessment to plan formulation. These findings might indicate that specific changes in team coordination could contribute toward the facilitation of specific team adaptation phases, but that those phases differ per team. In addition, no coordination changes occurred during plan formulation phases for either team, but they did occur during all other annotated phases of team adaptation (except for the adaptive team's team learning). While the maladaptive team underwent a change in coordination during their brief team learning phase, the adaptive team did not. However, this change could also be due to the maladaptive team being close to failing the game level.

Further examination entailed the inclusion of team events. Team events were shown as red points on the %DET line in Fig. 5, which are accompanied by a team event category code. Each number represents a different team event category, and a brief description can be found in Plot b of Fig. 5. An assessment of team coordination change in relation to team events revealed that in all cases where either team managed to shoot two buttons simultaneously (1a‐d), the events were preceded by a change in coordination. Moreover, after the maladaptive team encountered the first and second wave of enemies (2a‐b), multiple changes in coordination were identified, after which the team beat these enemy waves (3a‐b). However, only one change in coordination took place after encountering enemy wave three (2c), this team was not able to beat this wave of enemies (4a) and subsequently failed to complete the level. These results could indicate that appropriate amounts of change within low‐level team cognition contributed to the adaptation that was necessary for the maladaptive team's joint achievements. The adaptive team's data (Plot b) reveal another pattern. Relative to the maladaptive team's enemy waves, the adaptive team's waves were completed quicker. For a majority of these quickly completed enemy waves (70%), only one change in team coordination was identified. No changes occurred during the other 30% (2e‐3d, 2g‐3f, 2l‐3k). Contrary to the maladaptive team, this team had previously divided tasks during plan formulation phases, and might not have required change within low‐level team cognition to succeed during every enemy wave.

High‐level team cognition changes assessed through phases of team adaptation were also studied in relation to team events. Fig. 5 shows that team events mainly took place during regular gameplay (1a, 2a‐k, 2m, 3c‐l), or very close to a change from the plan execution phase to gameplay (1b‐d, 3a‐b). After the maladaptive team first encountered enemy waves that they successfully addressed (2a‐b), they went through plan formulation and execution phases. The adaptive team did not go through these adaptation phases to complete enemy waves. When the maladaptive team encountered wave three (2c), which they did not survive, they formulated a plan, but did not reach the plan execution phase. These results suggest that for the maladaptive team, plan formulation and execution could be triggered by team events, whereas those phases are not essential for the adaptive team with predetermined task divisions. Moreover, during enemy waves, no team learning occurred for the maladaptive team. Only after they failed the level, this phase was reached. The adaptive team did achieve team learning during waves. This could indicate that timely team learning might play an important role in successful team adaptation.

Lastly, we took into account the combination of change within team cognition at different levels, and team events. The first and second enemy waves that the maladaptive team completed successfully (3a‐b) took place during plan execution phases entailing one or more changes in team coordination, which were preceded by brief plan formulation phases. A similar pattern was identified for the adaptive team's simultaneous button press events (1c‐d). In addition, no coordination changes and events occurred during the plan formulation phases, whereas they did occur during all other types of annotated team adaptation phases. Our findings could indicate that teams self‐organize into patterns across multiple levels of team cognition to address demands, which is a key property suggested by the dynamical hypothesis. In other words, self‐organization into changes at multiple levels of team cognition could play a role in successfully achieving joint goals that heavily relied on coordination between team members.

## 4 Discussion

The current paper introduces an investigative framework to assess change in team cognition at different levels, under the dynamical hypothesis. By taking key properties of dynamical systems into account, as well as team events that relate to the high‐level team cognition variable of interest that potentially relates to change in team coordination, we provide a valuable additional layer to study team cognition. Consequently, our framework contributes theoretically and methodologically to the knowledge crucial to gaining a deeper understanding of team cognition, which can further enable the support of high‐level cognitive team processes and states based on low‐level team coordination dynamics. The steps within our framework include identifying the team cognition variables of interest (Step 1), examining their trajectories and visualizing changes between different phases of a high‐level team cognition process (Step 2), identifying change within low‐level team cognition and locating team events (Step 3). Finally, we connect the information gathered from previous steps, to jointly assess change at different levels of team cognition and team events (Step 4).

Findings obtained from applying the framework to contrasting cases (preliminarily) indicate that the maladaptive and adaptive teams’ low‐level cognition trajectories are dissimilar, with differences in general trends, and %DET value ranges. In addition, for team adaptation (i.e., high‐level cognition variable), trajectories start similarly with situation assessment following the external perturbation. After, teams diverge into different team adaptation phases. Moreover, when combining low‐ and high‐level cognition trajectories and team events, patterns of change differed between teams. The adaptive team exhibited less changes in team coordination and team adaptation phases than the maladaptive team, and displayed timely team learning throughout the game level. These findings might indicate that specific changes in team coordination and patterns in team adaptation phases contribute toward unsuccessful or successful team adaptation. While broader conclusions cannot be drawn due to the case study approach, these findings do show an example of the types of insights that can be generated regarding team cognition as a dynamical system, and they form testable predictions (see Table 3) that could be evaluated with a larger sample (Flyvbjerg, 2011).

**Table 3:** Examples of testable predictions that could be evaluated with a larger sample

a)	Change in low‐level team coordination precedes change in high‐level team adaptation phases
b)	Maladaptive teams require more change in team coordination to successfully adapt than adaptive teams
c)	Maladaptive teams change team adaptation phases after team events, adaptive teams do not
d)	Change in both team coordination and team adaptation phases precedes the achievement of joint goals that require coordination between team members

Moreover, the framework and its practical implementation also contributed to insights regarding the functionality of the dynamical hypothesis to assess team cognition. Several key properties of dynamical systems can be assessed with our framework. For example, our framework enables the examination of multistability within coordination dynamics (Kelso, 2012), and the visualization of change of the system across multiple levels over time (van Gelder & Port, 1995). In addition, through the assessment of change at multiple levels of team cognition in combination with team events, our findings indicate that teams self‐organize into patterns across multiple levels of team cognition to address a task demand, consistent with the dynamical hypothesis. In the case of our application, self‐organization into changes at multiple levels of team cognition could play a role in successfully achieving joint goals that heavily rely on coordination between team members.

However, a limitation of our current framework application is that data of only two teams were included. To provide generalizable conclusions regarding maladaptive and adaptive teams, future studies should include a larger number of teams. Yet, our examples could already be useful for researchers who are interested in qualitative insights from a specific team. Subsequently, we envision the application of our framework as two‐fold. First, when applied to a small sample of data, researchers can obtain idiographic insights and generate preliminary findings that inspire examinations on a larger scale. Second, when applied to a larger data sample, researchers can utilize our framework to test generalizable predictions regarding change in team cognition. Nevertheless, we hope that through the application of this investigative framework, our work inspires researchers to further assess change in team cognition through the lens of the dynamical hypothesis.

Overall, our study contributes to further integrating the dynamical hypothesis with team cognitive science in multiple ways: (1) the provision of a framework to assess team cognition through the lens of the dynamical hypothesis, and (2) the empirical application of the framework on team cognition data to not only illustrate the framework steps, but also assess the insights from the dynamical hypothesis regarding multiple levels of team cognition. Such contributions are important for understanding how teams operate and could form the foundation for augmenting teamwork in critical situations.

## Author contributions

Kyana van Eijndhoven: conceptualization, data curation, formal analysis, investigation, methodology, software, visualization, original draft preparation, reviewing, and editing. Travis J. Wiltshire: conceptualization, methodology, supervision, original draft preparation, reviewing, and editing. Elwira A. Hałgas: investigation and reviewing. Josette M. P. Gevers: conceptualization, supervision, and reviewing.

## Open Research Badges

This article has earned Open Data and Open Materials badges. Data and materials are available at https://osf.io/tsxcq and osf.io/x2f56.
